# How Do Individuals With Bipolar Disorder Experience Ecological Momentary Assessment and Mood Monitoring? A Systematic Review and Qualitative Meta-Synthesis

**DOI:** 10.1192/bjo.2025.10210

**Published:** 2025-06-20

**Authors:** Daljit Purewal, Georgina Shajan, Goldie Momoh, Shireen Patel, Laurence Astill Wright

**Affiliations:** 1 Institute of Mental Health, Nottingham, United Kingdom.; 2 NIHR ARC East Midlands, Nottingham, United Kingdom.; 3 Nottingham NIHR Biomedical Research Centre, Nottingham, United Kingdom.; 4 NIHR MindTech Medical Technology Collaborative, Nottingham, United Kingdom.; 5 Centre for Academic Mental Health, Bristol, United Kingdom

## Abstract

**Aims:** Advancements in smartphone technology and wearable devices allow for novel ways to monitor behaviour, mood, and mental state, as well as to develop new interventions. Understanding the perspectives and preferences of individuals with bipolar disorder (BD) is essential for the success of these mood monitoring interventions and for Ecological Momentary Assessment (EMA) as a data collection method.

This systematic review and meta-synthesis aimed to explore the user experience of mood monitoring and EMA, including the barriers and facilitators for both individuals with BD and clinicians, as well as the intended purposes of these tools.

**Methods:** A systematic review and meta-synthesis of qualitative studies were conducted (PROSPERO: CRD42023396473), focusing on the experiences of participants, users, and clinicians with mood monitoring and EMA in BD. Eight electronic databases were searched, and mixed-methods studies were included. A meta-synthesis approach was used to analyse the data, employing first-, second-, and third-order constructs, guided by Noblit & Hare's meta-ethnography framework. Studies were assessed for the risk of bias in qualitative research. Results were checked for coherence by individuals with lived experience and psychiatrists.

**Results:** The search identified 23,515 papers. A total of 20 studies using 12 different EMA protocols were identified and included in the meta-synthesis, from which nine overarching themes emerged: adverse effects, barriers to mood monitoring, facilitators of mood monitoring, the purpose of mood monitoring, negative experiences of data sharing, positive experiences of data sharing, clinician-related barriers and concerns, clinician-related facilitators and suggestions, and desired features.

**Conclusion:** This review highlights key factors that can enhance user experience, engagement, retention, usability, and acceptance of EMA and mood monitoring protocols for individuals with BD. A central finding is that users strongly value control over their data, with an emphasis on customisability and personalisation. Many users were sceptical about involving formal mental health services and preferred to use the tool as an aid for self-managing their condition in highly personal and iterative ways. We also report key adverse effects experienced by individuals with BD when engaging in mood monitoring, which may need to be addressed by incorporating additional therapeutic elements into the intervention — for example, subjective worsening of mood and the monitoring process serving as an unhelpful reminder of their mental illness.

